# An open source plant kinase chemogenomics set

**DOI:** 10.1002/pld3.460

**Published:** 2022-11-25

**Authors:** Maria Florencia Ercoli, Priscila Zonzini Ramos, Rashmi Jain, Joseph Pilotte, Oliver Xiaoou Dong, Ty Thompson, Carrow I. Wells, Jonathan M. Elkins, Aled M. Edwards, Rafael M. Couñago, David H. Drewry, Pamela C. Ronald

**Affiliations:** ^1^ Department of Plant Pathology and the Genome Center University of California Davis CA USA; ^2^ Centro de Química Medicinal (CQMED), Centro de Biologia Molecular e Engenharia Genética (CBMEG) Universidade Estadual de Campinas (UNICAMP) Campinas SP Brazil; ^3^ Structural Genomics Consortium (SGC) UNC Eshelman School of Pharmacy, University of North Carolina at Chapel Hill (UNC‐CH) Chapel Hill NC USA; ^4^ Division of Chemical Biology and Medicinal Chemistry UNC Eshelman School of Pharmacy, UNC‐CH Chapel Hill NC USA; ^5^ Centre for Medicines Discovery University of Oxford Oxford UK; ^6^ Structural Genomics Consortium University of Toronto Toronto Canada

**Keywords:** compound screening, ligation‐independent cloning, *Oryza sativa*, plant kinases, protein production, thermal shift assay

## Abstract

One hundred twenty‐nine protein kinases, selected to represent the diversity of the rice ( *Oryza sativa* ) kinome, were cloned and tested for expression in *Escherichia coli* . Forty of these rice kinases were purified and screened using differential scanning fluorimetry (DSF) against 627 diverse kinase inhibitors, with a range of structures and activities targeting diverse human kinases. Thirty‐seven active compounds were then tested for their ability to modify primary root development in Arabidopsis. Of these, 14 compounds caused a significant reduction of primary root length compared with control plants. Two of these inhibitory compounds bind to the predicted orthologue of Arabidopsis PSKR1, one of two receptors for PSK, a small sulfated peptide that positively controls root development. The reduced root length phenotype could not be rescued by the exogenous addition of the PSK peptide, suggesting that chemical treatment may inhibit both PSKR1 and its closely related receptor PSKR2. Six of the compounds acting as root growth inhibitors in Arabidopsis conferred the same effect in rice. Compound RAF265 (CHIR‐265), previously shown to bind the human kinase BRAF (B‐Raf proto‐oncogene, serine/threonine kinase), also binds to nine highly conserved rice kinases tested. The binding of human and rice kinases to the same compound suggests that human kinase inhibitor sets will be useful for dissecting the function of plant kinases.

## 1 INTRODUCTION

Protein phosphorylation is the most common form of posttranslational modification used in signal transduction by eukaryotic cells. In plants, protein kinases regulate key biological responses, such as hormone levels, metabolism, morphology, growth, and development (Bhargava & Sawant, 2013; Danquah et al., 2014; Deprost et al., 2007; Garcia et al., 2012; Marshall et al., 2012; Osakabe et al., 2013; Todaka et al., 2015; Wang et al., 2010; Wierzba & Tax, 2013; Wu & Cheng, 2014). As in other eukaryotes, protein kinases constitute one of the largest protein families within plant genomes. In rice (*Oryza sativa*), there are about 1500 genes that encode for recognizable protein kinase domains (~3.5% of the rice genome), the vast majority of which remain uncharacterized (Chandran et al., 2016; Goff et al., 2002; Manning et al., 2002; Yamamoto et al., 2012; Yu et al., 2002).

Genetic approaches, such as gene knockouts, have successfully identified plant kinases that mediate important traits but can be confounded by the fact that many plant genes have functionally redundant paralogues (Hicks & Raikhel, 2009). As a result, more than 40% of the genes in a plant genome are “invisible” to single knockout genetic screens. In addition, genes that cause lethality when knocked out cannot be discovered in these screens. This gap presents an opportunity for basic and applied science.

An alternative approach to genetic manipulation is to use a chemical biology strategy based on small molecule modulators of protein kinase function (Hicks & Raikhel, 2009, 2014). Protein kinases share similar ATP‐binding sites, and it is not uncommon for small molecule kinase inhibitors to be active against multiple, closely related kinases, suggesting that a kinase inhibitor may chemically “knockout” paralogues or even small families of kinases. Thus, using sets of carefully selected, well‐characterized kinase inhibitors that cover most of an organism's kinome in phenotypic assays allows the observed biological effect to be narrowed down to a small number of kinases (Uitdehaag et al., 2012). For human proteins, the construction of such a kinase chemogenomic set has allowed this strategy to successfully illuminate new biology and discover new therapeutic opportunities (Al‐Ali et al., 2015; Burdova et al., 2019; Jones & Bunnage, 2017; Wells et al., 2021). A similar approach has also been used to perform cost‐effective, chemistry‐based synthetic lethal screens in plants (Hicks & Raikhel, 2009, 2014; Xuan et al., 2013). Nevertheless, the lack of well‐characterized small molecule reagents has limited the exploration of plant kinomes.

Establishing a well‐characterized, broadly distributed Rice Kinase Chemogenomic Set would allow the scientific community to explore the function of rice kinases and deepen our understanding of plant signaling pathways. This endeavor would require the recombinant production of soluble, active rice kinases, the establishment of high‐throughput screening (HTS) assays to identify small molecule ligands from libraries of compounds, and iterative chemistry to optimize compound selectivity profiles. These compounds would then be used in phenotypic screens to investigate the biological impact of modulating the function(s) of the target kinase(s). The on‐target activity of inhibitors that confer interesting phenotypes could then be verified via chemoproteomics and further validated using genetic tools, such as the creation of rice knockout lines (Huber & Superti‐Furga, 2016). Broad distribution would allow the community to use this compound set in a range of phenotypic assays relevant to different facets of plant biology.

Importantly, the conservation of the overall protein kinase architecture, biochemical activity, and ATP‐binding site across distantly related species should allow the knowledge, protocols, assays, and reagents obtained during the development of the human kinase chemogenomic set to be used in the establishment of a similar set of reagents for rice kinases. Indeed, it is now well established that small molecule inhibitors originally designed for human kinases are also active against kinases from unrelated organisms, such as eukaryotic parasites and plants (Alam et al., 2019; Aquino et al., 2017; Peña et al., 2015). Likewise, the strategy to combine available structural information with high‐throughput cloning adopted by structural genomics initiatives to expedite the recombinant production of soluble, active human proteins (Savitsky et al., 2010) has also been shown effective for plant proteins (Tosarini et al., 2018). Finally, HTS assays used to identify ligands for human proteins (Niesen et al., 2007) have been applied with success for plant protein kinases (Aquino et al., 2017).

Here, we established the groundwork for the creation of a Rice Kinase Chemogenomic Set and identified a previously unknown connection between 16 compounds and primary root length. We also show that one compound, previously shown to bind the human kinase BRAF (B‐Raf proto‐oncogene, serine/threonine kinase), also binds at least to nine rice kinases. Our data thus suggest that the methods used for the generation of the human kinase chemogenomics set are readily applicable to dissecting kinase function in plants. Further, we show that small molecule kinase inhibitors can be used to identify new biological processes, contributing to the development of knowledge that will be of interest to the wider plant science community.

## 2 RESULTS

### 2.1 Selection of protein kinases

**FIGURE 1:**
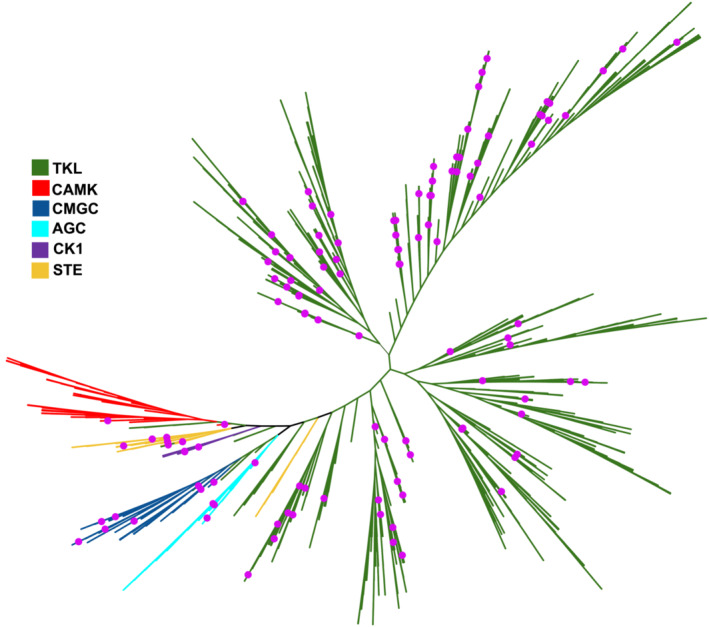
A phylogenetic tree showing the rice kinases selected for this study (pink dots). The rice kinome contains 1467 proteins that are classified into six kinase groups: TKL (Tyrosine Kinase‐Like), green; CAMK (Ca^2+^/calmodulin‐dependent protein kinase), red; CMGC (cyclin‐dependent kinase [CDK], mitogen‐activated protein kinase [MAPK], glycogen synthase kinase [GSK], and CDC‐like kinase [CLK]), blue; AGC (AMP‐dependent kinases [PKA], cGMP‐dependent kinases, and the diacylglycerol‐activated/phospholipid‐dependent kinase PKC), cyan; CK1 (Casein kinase 1), purple; and STE (Sterile serine/threonine kinases), saffron. The phylogenetic tree was constructed using the unweighted neighbor‐joining method and drawn using Interactive Tree Of Life (iTOL) v5 online tool (Letunic & Bork, 2021).

The rice genome has 1467 genes encoding a recognizable protein kinase domain. These can be divided into 63 distinct kinase families belonging to six kinase groups (AGC, CAMK, CK1, CMGC, STE, and TKL) based on sequence identity levels as established by the rice kinase phylogenomics database (Dardick et al., 2007; Jung et al., 2015) (Figure 1). To select a representative set of protein kinase genes from the rice genome, we first checked expression values of these genes in 21 available RNA‐Seq libraries from the Rice Genome Annotation Project database containing data from samples collected from various rice tissues during different developmental stages or under various biotic and abiotic stresses (Kawahara et al., 2013). We selected 975 genes having expression levels ≥ 2.0 from this analysis.

We next employed RICENet v2, a probabilistic gene network to enrich for trait‐associated genes among the selected 975 rice protein kinase‐encoding genes (Lee et al., 2011, 2015). This analysis resulted in the selection of 141 kinase‐encoding genes representing 45 out of the 63 kinase families predicted to participate in independent pathways. Then, we selected one kinase‐encoding gene from each of the remaining 18 kinase families to ensure that at least each kinase family was represented by at least one member. Finally, we also included in our set three well‐studied kinase‐encoding genes: the kinase domain of the rice disease resistance gene XA21(AAC49123) (Song et al., 1997), the XA21‐coreceptor (OsSERK2, LOC_Os04g38480) (Chen et al., 2014), and a histidine kinase (LOC_Os06g44410) (Taylor et al., 2021) known to regulate rice root development. Thus, the initially selected set consisted of 162 genes. We further predicted domain information of these kinases using Pfam (Mistry et al., 2021). Out of the 162 selected rice genes, we removed 15 whose gene products lacked a predicted full kinase domain and thus are unlikely to bind inhibitors. Among the remaining genes, we could not obtain synthetic DNA for 18 due to gene synthesis failure (including the histidine kinase LOC_Os06g44410). Following subtraction of these genes, the final set consisted of 129 rice kinases representing diversity within the rice kinome that were successfully synthesized (Figure 1 and Data Set S1).

### 2.2 Recombinant production of selected rice protein kinases

Heterologous expression of eukaryotic genes in a bacterial host may lead to the production of insoluble or inactive recombinant protein. Here, we adopted a high‐throughput, protein structure‐based strategy to quickly identify protein constructs that can be recombinantly produced in a soluble form in *Escherichia coli* (Savitsky et al., 2010; Tosarini et al., 2018). For each of the 129 selected rice protein kinase genes, we designed an average of four different constructs for expression of the isolated kinase domain with varying N‐ and C‐termini. Construct design was based on the best matches from the Protein Data Bank (PDB) for each of the selected rice kinases, identified using the PSIPRED server (Buchan & Jones, 2019). DNA fragments representing each of these kinase domain truncations were obtained via polymerase chain reaction (PCR) using the appropriate set of synthetic DNA template and oligonucleotide primers (see Data Set S1). Amplicons were cloned via ligation‐independent cloning (LIC) into a pET28‐based expression vector, which added a cleavable 6xHis tag to the N‐terminus of the recombinant protein (Aslanidis & de Jong, 1990; Stols et al., 2002; Strain‐Damerell et al., 2014). In total, 515 constructs, representing all 129 selected rice kinase‐encoding genes, were successfully cloned (see Data Set S1).

Soluble recombinant production of all 515 rice kinase constructs in two different *E. coli* strains was evaluated using small‐scale test expression (1 ml cultures) followed by purification via ion metal affinity chromatography (IMAC, facilitated by the presence of the N‐terminal 6xHis tag in the recombinant protein) from clarified cell lysates. IMAC eluates were visualized by denaturing sodium dodecyl sulfate polyacrylamide gel electrophoresis (SDS‐PAGE). These analyses revealed that 286 of the 515 constructs (55.5%) could be purified from clarified cell lysates, as indicated by the presence of a protein band of the expected molecular weight (see Data Set S1 and Figures S1 and S2). Overall, we could detect the soluble production of 85 out of the 129 selected rice protein kinases (66%). Forty of these protein kinases were then purified in milligram scale for chemical screening studies.

### 2.3 Ligand identification

To identify ligands for the purified rice kinases from a library of commercially available human kinase inhibitors, we used a thermal‐stability assay (differential scanning fluorimetry [DSF]). This assay is based on the ability of a ligand to stabilize a target protein and increase its temperature‐induced unfolding midpoint (*T*_m_) compared with a no‐ligand control (reported as a Δ*T*_m_). DSF has been extensively employed to assess binding of compounds to target protein kinases and to estimate compound promiscuity (Elkins et al., 2016; Fedorov et al., 2012). Compound library selection took into account three main criteria. First, all compounds used here are readily available from commercial vendors. This makes it easy to obtain compounds for follow‐up phenotypic assays in plants, which are likely to use large quantities of material. Second, the 627 compounds included in our library have a wide range of chemical scaffolds. As the development of plant kinase inhibitors is still in its infancy, we opted to use a library with a large chemical diversity. Finally, compounds in our library target a wide range of human kinases having diverse biological functions (see Data Sets S2 and S3).

**FIGURE 2:**
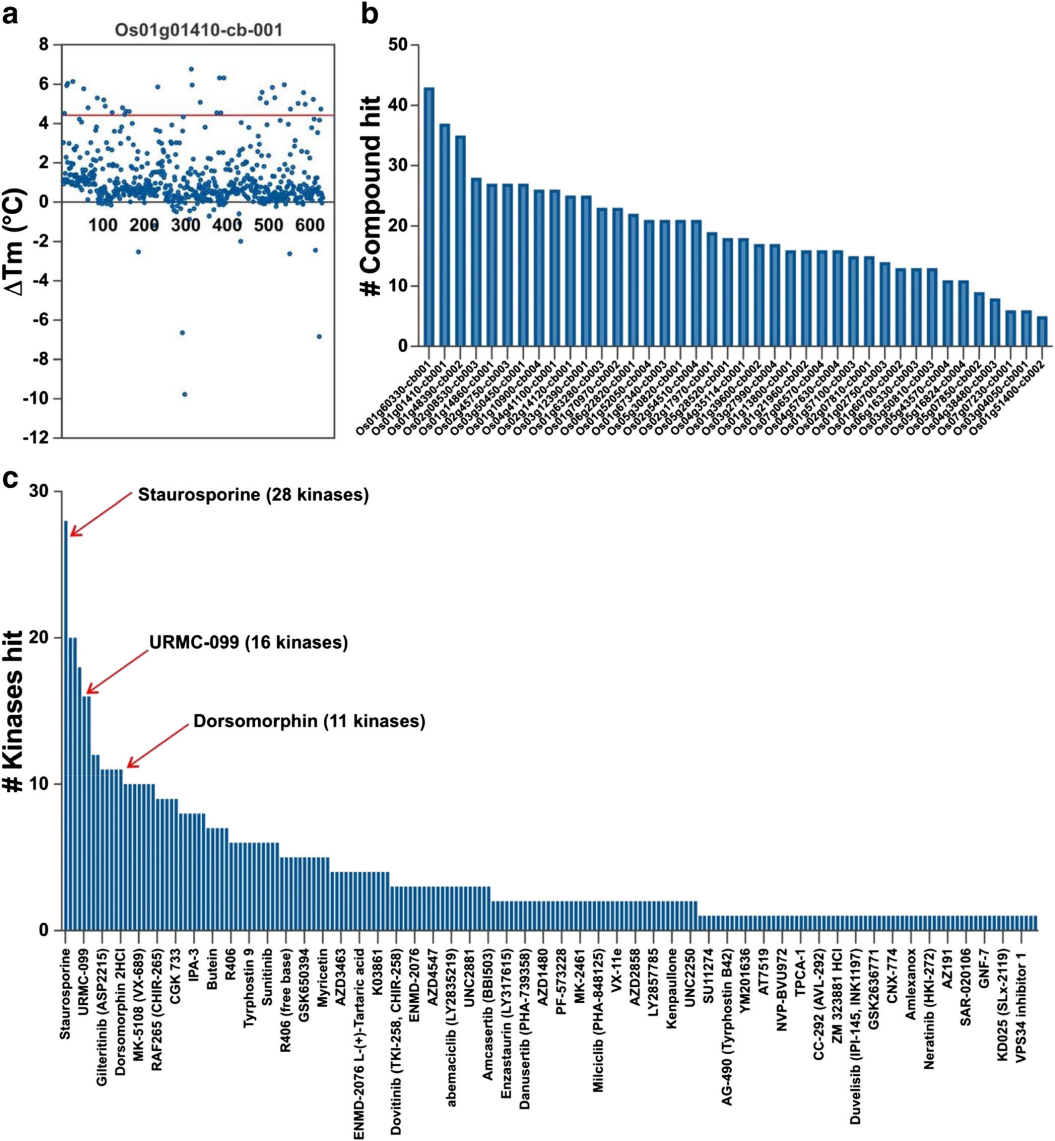
Screening kinase inhibitors against a subset of rice kinases. (a) Example thermal shift data set from screening of 627 compounds against Os01g01410‐cb‐001. Most compounds show no stabilization of the protein, with thermal shift (Δ*T*_m_) values near 0°C. The red line marks 2x the standard deviation of the dimethyl sulfoxide control, and hits are defined as compounds that lead to a temperature shift at or above this threshold. (b) This bar chart depicts the number of compounds that are classified as hits in the Δ*T*_m_ assay for each rice kinase screened. Kinases to the left bind many different compounds, whereas kinases to the right bind only a few of the molecules in the screening set. (c) This bar chart provides an indication of the promiscuity of these compounds against this panel of rice kinases. More than a dozen of these compounds (left portion of the bar chart) stabilize 10 or more kinases in the panel, indicating that they are relatively promiscuous, or non‐selective. Three of these compounds that are also known to be promiscuous against the human kinome are marked.

Using DSF, we collected temperature denaturation curves for 40 purified kinases in the presence of each one of the 627 compounds in our library (plus vehicle, dimethyl sulfoxide [DMSO]; and positive, staurosporine; control). A complete matrix of the thermal shift data is available in Data Set S2; Data Set S3 provides a different visualization of this matrix in which the thermal shift data were sorted for each kinase separately. A hit was defined as a compound that increased thermal stabilization at least 2x the standard deviation of the DMSO control (Chilton et al., 2017). An example plot of the data and hit identification is depicted in Figure 2a for Os01g01410‐cb‐001.

As expected, the overall results mirror previous experiments that interrogated a panel of human kinases with a set of kinase inhibitors (Bamborough et al., 2008; Elkins et al., 2016; Fedorov et al., 2012; Posy et al., 2011). In “all versus all” screens, one often identifies promiscuous compounds that bind to many targets, selective compounds that bind very few targets, promiscuous kinase targets that bind a variety of chemotypes, and kinase targets that are more difficult to inhibit and bind relatively few structural classes of inhibitors. Hit rates ranged from a high of 6.8% for Os01g60330‐cb001 (43 hits) to a low of .8% for Os01g51400‐cb002 (5 hits) (Figure 2b).

Figure 2c shows the compounds that qualified as a hit for at least one kinase in the panel, sorted by the number of kinases hit. Three promiscuous human kinase inhibitors (staurosporine, dorsomorphin, and URMC‐09928) are highlighted that also demonstrate promiscuous binding in this small rice kinase panel. Twenty‐eight of the 40 rice kinases showed significant stabilization with staurosporine, a very promiscuous human kinase inhibitor. Twenty of the compounds stabilized (implying a binding event) 10 or more of these rice kinases screened (Figure 2c). Four hundred sixteen of the compounds did not significantly stabilize any of these rice kinases.

Finally, a number of FDA‐approved kinase inhibitors are in this screening set, and many show binding to at least one rice kinase (see Data Set S4). Some FDA‐approved medicines, such as gilteritinib, sunitinib, and vemurafenib, stabilize five or more of these rice kinases. A number of quite selective human kinase inhibitors such as the ERBB2 inhibitor lapatinib, EGFR inhibitor gefitinib, and MEK inhibitors trametinib and cobimetinib did not stabilize any of the rice kinases screened.

### 2.4 Kinase inhibitors affect primary root development in Arabidopsis and rice

From the set of rice kinase inhibitors that were identified by DSF, we selected a group of 37 compounds and tested them for their ability to affect plant development. This subset was chosen to include promiscuous inhibitors targeting several kinases simultaneously and compounds that specifically bind a small group of kinases (Data Sets S2 and S3). Some of the rice kinases that these compounds bind and likely inhibit include orthologues of Arabidopsis kinases as BRASSINOSTEROID INSENSITIVE 1 (BRI1) (Friedrichsen et al., 2000; Hacham et al., 2011; Kang et al., 2017; Li & Chory, 1997), SOMATIC EMBRYOGENESIS RECEPTOR‐LIKE KINASE 1 and 2 (SERK1 and SERK2) (Du et al., 2012; Gou et al., 2012), CYCLIN‐DEPENDENT KINASE F;1 (Takatsuka & Umeda, 2014), and PHYTOSULFOKIN RECEPTOR 1 (PSKR1) (Matsubayashi et al., 2002, 2006), which are known to control root development. Therefore, we decided to first test the activity of these compounds based on their ability to modify the primary root development using Arabidopsis.

**FIGURE 3:**
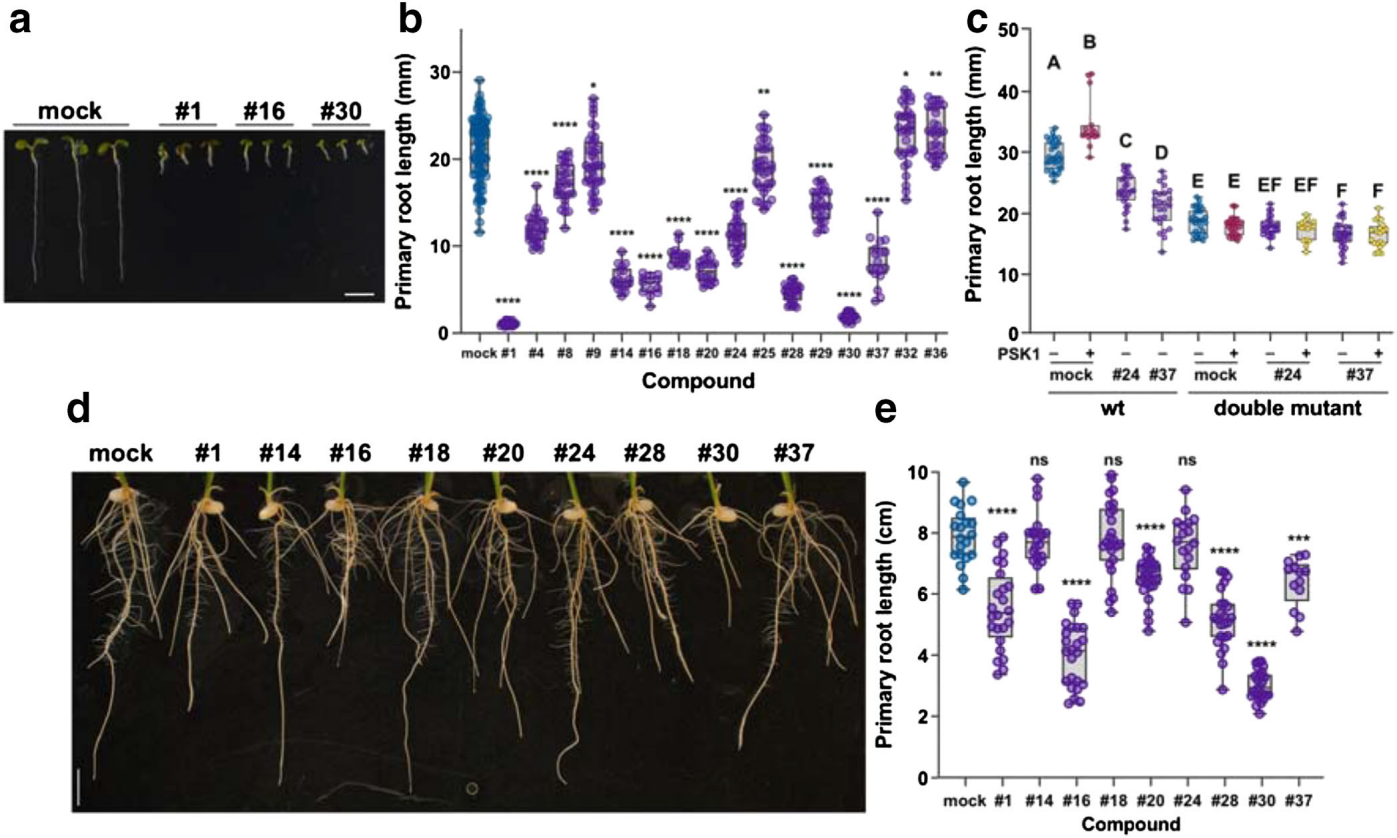
Human kinase inhibitors modify primary root development in Arabidopsis and rice. (a) Root growth phenotype 6 days after sowing of Col‐0 (wt) seedlings grown on 1xMS vertical plates with or without 1 μM of the selected kinase inhibitor showing a significant effect on primary root growth. (b) Primary root length (mm) 6 days after sowing of wt seedlings grown on 1xMS vertical plates prepared with or without 1 μM of the selected kinase inhibitor. (c) Primary root length (mm) 7 days after sowing of wt and double mutant (*Atpskr1, Atpskr2*) seedlings grown on 1xMS vertical plates prepared using different combinations of two selected kinase inhibitors that are known to bind the rice orthologue of AtPSKR1 (#24 and #37, 1 μM) and PSK1 (100 nM). (d) Root growth phenotype and (e) primary root length (cm) 7 days after sowing of kitaake seedlings grown on 1xMS vertical plates with or without 1 μM of the selected kinase inhibitor. The data shown in (b), (c), and (e) are a box and whisker plot combined with scatter plots; each dot indicates an individual measurement (*n* = 20–30). In (b) and (e), *p* values are calculated by two‐tailed Student's *t*‐test (*p ≤ .05, **p ≤ .01, ***p ≤ .001, ****p ≤ .0001). In (c), different letters indicate significant differences, as determined by ANOVA followed by Tukey's multiple comparison test (*p* < .05).

Of the 37 compounds tested, 14 caused a significant reduction of primary root length (Figures 3a,b and S3A,B) and only 2 produced a mild increase of elongation compared with the control plants (Figure 3b).

We found that compounds #24 (Hesperadin) and #37 (Sitravatinib) each bind the rice kinase (LOC_Os04g57630), which is predicted to be an orthologue of Arabidopsis PSKR1 (AtPSKR1) (see Data Set S2), the main receptor of PSK, a small sulfated peptide that positively controls root development (Matsubayashi et al., 2006; Matsuzaki et al., 2010) (Figure 3c). *pskr1* is phenotypically indistinguishable from WT and still responds to synthetic peptide treatment due to the presence of a second PSK receptor (AtPSKR2) that shares almost 50% sequence identity with AtPSKR1. The double mutant (*Atpskr1, Atpskr2*) has shorter roots and is insensitive to PSK treatment (Amano et al., 2007). The short root phenotype observed after chemical treatment may be a consequence of the inhibition of both PSKR1 and PSKR2 (Figure 3b,c). In support of this hypothesis, we observed that exogenous addition of the PSK peptide did not rescue the short root growth phenotype of WT plants treated with compound #24. In contrast, a significant response was obtained when combined with compound #37, although the effect was not enough to complement the phenotype of untreated WT (Figure S3C). It is possible that the lack of response to PSK treatment is a consequence of the inhibition of a different set of kinases, which have a detrimental effect on root growth that is not rescued by PSK treatment. To further test this hypothesis, we performed the same treatments using the double mutant (*Atpskr1, Atpskr2*). When treating the mutant with compound #24, we saw no further reduction in primary root length (Figure 3c). Compound #37, on the other hand, produces a slight inhibition of the mutant root growth (Figure 3c), though this inhibition is not as strong as that observed when treating control plants. Together, these results indicate that these chemicals may be totally (compound #24) or partially (compound #37) inhibiting the kinase activity of AtPSKR1 and AtPSKR2.

We next tested the response of rice primary root development to the compounds showing the most significant effects in Arabidopsis. From the nine compounds tested, six caused a reduction in rice primary root development (Figure 3d,e).

Three compounds significantly affected Arabidopsis and rice seedling development (#1, #16, and #30) (Figure 3a,b,d,e). Compound #1, staurosporine, stabilizes 28 kinases in our panel, compound #16 (AD80) stabilizes 10 kinases, and compound #30 (PIK‐75) stabilizes 6 kinases (Data Sets S2 and S3).

Overall, these results indicate that some of the human kinase inhibitors that can interact with plant kinases based on DSF cause a modification in root growth. Further work is needed to establish structure–activity relationships for individual kinases, verify inhibition of kinase activity in the plant, and build our understanding of the consequences of poly‐pharmacology (inhibition of multiple kinases by one compound) on phenotype.

### 2.5 Multiple sequence alignments suggest that compound RAF265 targets similar regions in human and rice kinases

We next compared the BRAF human kinase with nine rice kinases stabilized by the same compound RAF265 (CHIR‐265). Multiple sequence alignment using the online tool Clustal Omega (v 1.2.4) revealed that all 11 subdomains indicative of a protein kinase (Hanks et al., 1988) are conserved in the nine rice kinases and the BRAF human kinase (Figure S4). Strikingly, subdomain VI, containing the HRD motif important for catalysis and ending in an invariant Asn involved in substrate binding, is particularly well conserved. Furthermore, examination of the BRAF residues involved in binding compound RAF265 according to the co‐crystal structure in the PDB (ID 5CT7) (Williams et al., 2015) reveals that these residues are generally highly conserved in the nine rice kinases (Figure S4). These results suggest that RAF265 inhibits the function of both plant and animal kinases in the same manner, as an ATP competitive inhibitor.

## 3 DISCUSSION

Chemical biology refers to the use of small molecules that can act as agonists/antagonists to specifically activate/block the function of a protein or members of a protein family. Using chemical probes provides an exciting alternative to overcoming the problems of gene redundancy, lethality, and pleiotropy that frequently hinder gene function studies. Another advantage of chemical biology is that perturbations to the system under study can be temporary because these compounds can be applied conditionally, reversibly, and dose‐dependently during a specific developmental phase (Halder & Russinova, 2019).

Despite the fact that plant biologists have used chemical biology screening to identify compounds that affect relevant biological processes such as pattern‐triggered immunity (Bektas & Eulgem, 2015), trafficking routes (Drakakaki et al., 2011), plant cell wall properties (Brabham & DeBolt, 2013; Yoneda et al., 2007), and plant hormone signaling (De Rybel et al., 2009; Halder et al., 2019; He et al., 2011; Meesters et al., 2014; Park et al., 2009; Tsuchiya et al., 2010), the potential of plant chemical genetics research remains largely unexplored. The selection of an appropriate screening procedure and screening compound library is one of the first steps toward answering new questions from a multidisciplinary chemical genetic perspective. Using strategies developed in other organisms can help solve these problems when the targets are well conserved, such as in the case of protein kinases that share similar ATP‐binding sites across distantly related species.

In this work, we tested whether compounds from a chemogenomic set that had previously been shown to bind human kinases also bind rice kinases. To accomplish this, we first created constructs with appropriate domain boundaries and then determined optimal expression conditions to successfully produce recombinant proteins in a heterologous system (Savitsky et al., 2010; Tosarini et al., 2018). For each of the 129 proteins, we created an average of four different constructs with varying N‐ and C‐terminal boundaries encompassing the kinase domain. We performed a small‐scale test expression (1 ml cultures) in two different *E. coli* strains to identify the most promising constructs and expression conditions for protein production. Using this method, we were able to detect the soluble production of 85 of the 129 rice protein kinases (66%). To our knowledge, this is the first comprehensive study that reports DNA constructs and expression conditions for the soluble production of a representative set of rice protein kinases using an *E. coli* expression system.

We identify inhibitors of 40 rice kinases using a thermal‐stability assay (DSF). This is valuable information that can be used to develop not only more comprehensive chemogenomic compound sets for phenotypic screening but also tool compounds with specificity for particular plant kinase(s) that can be used to explore their functions. Although DSF is a technique with a very low false positive rate and there is a strong correlation between the observed change in protein melting temperature and the inhibitor binding affinity (Elkins et al., 2016; Fedorov et al., 2012), the absolute values for the inhibitor binding affinities were not measured. As a result, before embarking on a project based on specific kinase:inhibitor interactions, a preliminary step would be to confirm the binding affinity and/or inhibition of enzymatic activity of the targeted kinase. This limitation is less of a concern when using the inhibitor set in phenotypic screens to identify kinases that will then be validated using genetic tools because the goal of the experiment is to create a shortlist of candidate kinases.

Based on the effect on primary root elongation in Arabidopsis and rice, the ability of 37 active compounds to modulate a biological response in plants was tested. Two chemicals that inhibit root growth bind to the Arabidopsis PSKR1 orthologue, one of the two PSK receptors that positively regulate root development. The root growth of a double receptor kinase mutant that is insensitive to PSK does not respond to these chemical compounds, demonstrating how the results of the DSF screening could be further validated using genetic complementary approaches.

## 4 MATERIALS AND METHODS

### 4.1 Cloning of rice protein kinase domains into expression vector pNIC28‐Bsa4

Full‐length cDNA sequences for the selected rice kinases were de‐novo synthesized (the Sainsbury Laboratory) and used as the templates for PCR amplifications. Multiple fragments encompassing the kinase domains (KD) of these genes were amplified and cloned into expression vector pNIC28‐Bsa4 (GenBank Accession No. EF198106), using LIC (Aslanidis & de Jong, 1990; Burgess‐Brown et al., 2014; Gileadi et al., 2008; Stols et al., 2002). On average, four constructs were designed for each target KD, varying the N‐ and C‐terminal boundaries. T1 phage‐resistant *E. coli* Mach‐1 cells (Invitrogen, Carlsbad, CA, USA) were used for general cloning. Proteins cloned into pNIC28‐Bsa4 vector are fused to an amino‐terminal tag of 22 residues (MHHHHHHSSGVDLGTENLYFQ*SM), including a hexahistidine (His6) and a TEV‐protease cleavage site. LIC sites are separated by a “stuffer” fragment that contains the *Bacillus subtilis sacB* gene, which allows negative selection on agar plates containing 5% sucrose (Stols et al., 2002). PCR fragments were annealed to the linearized vector through complementary single‐stranded regions generated by the T4 DNA polymerase 3′‐exonuclease activity. Vector cloning sites were generated by cleavage at two sites by the restriction enzyme *Bsa*I, followed by T4 DNA polymerase treatment in the presence of dGTP. The inserts were treated in the presence of dCTP. Clones were screened by colony PCR and verified by DNA sequencing, using primers specific to the vector: pLIC‐F (5′‐TGTGAGCGGATAACAATTCC‐3′) and pLIC‐R (5′‐AGCAGCCAACTCAGCTTCC‐3′).

### 4.2 Small‐scale test expression

In order to generate expression clones, rice KD constructs were transformed into *E. coli* strains derived from BL21(DE3) and Rosetta 2 (Merck Millipore, Burlington, VT, USA), BL21(DE3)‐R3‐pRARE2 and BL21(DE3)‐R3‐lambda‐PPase. Strain BL21(DE3)‐R3‐pRARE2 is a phage‐resistant derivative of BL21(DE3) transformed with the pRARE2 plasmid from Rosetta 2 cells, which carries chloramphenicol resistance, whereas strain BL21(DE3)‐R3‐lambda‐PPase is a phage‐resistant derivative of BL21(DE3) transformed with a pACYC‐derived plasmid that expresses the bacteriophage‐lambda phosphatase as well as three rare tRNAs (Gileadi et al., 2008). Both strains were a kind gift of SGC Oxford. To find the best constructs and the optimal expression conditions for protein production, all positive clones were evaluated by small‐scale test expression followed by IMAC purification from clarified cell lysates. Small‐scale test expressions followed the 1‐ml expression system described previously (Burgess‐Brown et al., 2014; Savitsky et al., 2010). In summary, overnight cultures of expression clones were prepared in 1 ml of Lysogeny broth (LB) medium containing antibiotics (50 μg/ml kanamycin and 34 μg/ml chloramphenicol) in a 96‐well deep well block (Sarstedt), and cultures were grown overnight in a microplate shaker (Titramax 101, Heidolph) at 37°C. Overnight cultures (20 μl) were inoculated into 1 ml of Terrific broth (TB) medium containing only kanamycin (50 μg/ml) and incubated in a microplate shaker (Titramax 101, Heidolph) at 37°C, until an optical density at 600 nm (OD_600_) of 2–3. Then, expression was induced by adding 0.1 mM of IPTG, and cultures were incubated overnight in a microplate shaker (Titramax 101, Heidolph) at 18°C. Cells were harvested by centrifugation (3,500× ***g*** for 20 min) and suspended in 200 μl of lysis buffer (50 mM of 4‐(2‐hydroxyethyl)‐1‐piperazineethanesulfonic acid [HEPES], pH 7.5, 0.5 M of NaCl, 10% glycerol, 10 mM of imidazole, and 0.5 mM of tris‐(2‐carboxyethyl) phosphine hydrochloride [TCEP]), containing 0.1% dodecyl maltoside (DDM), protease inhibitor cocktail EDTA‐free (Cat. Number 539134, Merck Millipore; 1:200), 0.5 mg/ml lysozyme, and 50 units/ml benzonase. After freezing the cell suspensions at −80°C for 20 min, the block was placed in a water bath for approximately 15 min at room temperature, allowing slight thawing. Samples were mixed and an aliquot (3 μl) of the total lysate fraction was removed from each well for future analysis. The lysate was clarified by centrifugation (3,500× ***g*** for 10 min) and the supernatant collected in a fresh 96‐well deep well block and incubated with 25 μl of pre‐equilibrated Ni‐sepharose resin (GE Healthcare Life Sciences) in lysis buffer in a microplate shaker (Titramax 101, Heidolph) at 18°C for 1 h. The contents of each well were transferred to a 96‐well filter plate (Thomson); the resin was washed with 200 μl of wash buffer (50 mM of HEPES, pH 7.5, 0.5 M of NaCl, 10% glycerol, 30 mM of imidazole, and 0.5 mM of TCEP) and centrifuged at 300× ***g*** for 1 min. The wash procedure was repeated three more times. Finally, 40 μl of elution buffer (50 mM of HEPES, pH 7.5, 0.5 M of NaCl, 10% glycerol, 300 mM of imidazole, and 0.5 mM of TCEP) was added to each well and proteins were eluted from the resin by centrifugation at 300× ***g*** for 3 min. Eluted fractions were analyzed by SDS‐PAGE. The identity of the purified proteins was further confirmed by liquid chromatography‐mass spectrometry (LC‐MS).

### 4.3 Mid‐scale protein expression and purification

Protein expression and purification followed procedures previously described (Tosarini et al., 2018). Briefly, overnight starter cultures were grown in LB medium containing kanamycin (50 μg/ml) and chloramphenicol (34 μg/ml) in an incubator shaker at 37°C. Five milliliters of the starter culture was used to inoculate 500 ml of TB medium supplemented with kanamycin (50 μg/ml). Cells were cultivated at 37°C until an OD_600_ ~ 1.8. The culture was then transferred to an incubator shaker at 18°C. After a 30‐min cool‐down period, IPTG was added to a final concentration of 0.2 mM. Cells were further cultivated for 16 h at 18°C. Cells were collected by centrifugation (15 min, 6,000x ***g*** at 4°C). The pellet was suspended in 2x lysis buffer (1 ml/g of cells) (1x lysis buffer is 50 mM of HEPES, pH 7.5, 0.5 M of NaCl, 5.0% [v/v] glycerol, 10 mM of imidazole, and 1 mM of TCEP) supplemented with protease inhibitor cocktail EDTA‐free (Merck Millipore; 1:200). Cells were stored at −80°C until use. Cells were lysed by sonication (Sonics Vibra Cell VCX750 ultrasonic cell disrupter) on ice for 5 min (5 s on, 10 s off, amplitude = 35%). Polyethyleneimine (PEI, 5% [w/v], pH 7.5) was added to the cell lysate to a final concentration of 0.15%, prior to clarification by centrifugation (45 min, 40,000x ***g***, 4°C). Recombinant proteins were enriched from the clarified lysate by gravity‐flow IMAC. Chelating Sepharose Fast Flow resin (Cat. Number 17057502, GE Healthcare) was loaded with Ni^2+^ according to the manufacturer's instructions. A total of 3 ml of Ni^2+^‐loaded resin was packed into Econo‐Pac columns (Cat. Number 7321010, Bio‐Rad) and equilibrated with 3 column volumes (CV) of elution buffer (binding buffer supplemented with 300 mM of imidazole; binding buffer is 50 mM of HEPES, pH 7.5, 0.5 M of KOAc, 10% glycerol, 50 mM of arginine and glutamate, 10 mM of imidazole, and 1 mM of TCEP) and 5 CV of binding buffer. Fractions for the flow‐through, 10 mM of imidazole wash (in binding buffer, 10 CV), 30 mM of imidazole wash (in binding buffer, 5 CV), and 300 mM of imidazole elution (in binding buffer, 3 CV), were collected and analyzed by 12% SDS‐PAGE. Selected IMAC fractions were pooled together and dialyzed (MW cutoff 10 kDa) against excess gel filtration buffer (GF buffer is 10 mM of HEPES, 0.5 M of KOAc, 10% glycerol, 50 mM of Arg‐Glu, and 1 mM of TCEP). TEV protease (in a mass ratio of 1:10) was added directly to the dialysis bag. TEV protease treatment was performed overnight at 4°C. Recombinant proteins lacking the 6His tag were further purified via reverse IMAC using 0.8 ml of Ni^2+^‐loaded Chelating Sepharose Fast Flow resin packed into poly‐prep® chromatography columns (Cat. Number 7311550, Bio‐Rad) and prepared as above. Fractions for the flow‐through, 10 mM of imidazole wash (in GF buffer, 10 CV), 30 mM of imidazole wash (in GF buffer, 5 CV), and 300 mM of imidazole elution (in GF buffer, 3 CV), were collected and analyzed by 12% SDS‐PAGE. Reverse IMAC fractions containing the protein of interest were pooled together and concentrated to a final volume of 5.0 ml. Samples were clarified by centrifugation (10 min at 21,000x ***g*** and 4°C) and injected onto a pre‐equilibrated Hiload 16/600 Superdex 200 pg (in GF buffer) connected to an AKTA pure system (GE Healthcare) set at 0.8 ml/min. Protein samples were concentrated by centrifugation using spin columns (MW cutoff of 10 kDa) (Cat. Number UFC501096, Merck Millipore). Protein concentration was estimated by UV using calculated extinction coefficients. Protein samples were flash‐frozen in a liquid nitrogen bath and stored at −80°C until use.

### 4.4 Differential scanning fluorimetry

Small molecule screening by DSF was performed as described previously (Fedorov et al., 2012; Niesen et al., 2007). Briefly, the DSF assay was performed in the 96‐well format. Purified rice kinase protein was diluted to 2 μM of kinase in 100 mM of potassium phosphate, pH 7.5, 150 mM of NaCl, and 10% glycerol supplemented with 5 × SYPRO Orange (Invitrogen). All assay experiments used 19.5 μl of 2 μM of kinase and SYPRO Orange mixture. Compounds solubilized in DMSO were used at a 12.5‐μM final concentration, with a 2.5% concentration of DMSO per well. PCR plates were sealed using optically clear films and transferred to a C1000 thermal cycler with CFX‐96 RT‐PCR head (Bio‐Rad). The fluorescence intensity was measured over a temperature gradient from 25°C to 95°C at a constant rate of 0.05°C/s. Curve fitting and protein melting temperatures were calculated based on a Boltzmann function fitting to experimental data (GraphPad Prism 8). Protein with the addition of 2.5% DMSO was used as a reference. All experiments were carried out in triplicate, and the mean of the Δ*T*_m_ is reported. Compounds that provided negative values are presented as having a Δ*T*_m_ of 0°C.

### 4.5 Arabidopsis/rice seedling analysis

Seeds from *Arabidopsis thaliana* accession Col0 and double mutant (*Atpskr1, Atpskr2*, which also contains an additional insertion in gene At1g72300) (Amano et al., 2007) and from *Oryza sativa* ssp. *japonica* cultivar Kitaake were used in this study. For primary root analysis, Arabidopsis seeds were surface‐sterilized in 70% ethanol and then stratified in 0.1% agarose in the dark (4°C) for 2 to 3 days, whereas rice seeds were dehulled, surface‐sterilized in 20% bleach for 30 min, and then washed thoroughly with autoclaved water. The seeds were sown on a solid medium containing 1x Murashige and Skoog salt mixture and 1% sucrose (pH 5.8) in .3% Gellex (Gellan Gum CAS Number 71010‐52‐1, Caisson Laboratories) supplemented with or without 1 μM of the selected kinase inhibitor (see Data Sets S2 and S3 for a description of the compounds tested in this study). The inhibitors were stored as 10 μM stocks in DMSO. Plates containing DMSO were used as controls. Synthetic PSK1 is tyrosine‐sulfated and was obtained from Pacific Immunology (Ramona, CA, USA). The peptide was stored as 1 μM stocks in water ddH_2_O. The top half of the Petri dish was sealed with Micropore tape to allow gas exchange and plates were placed vertically for 6 days in chambers with 16‐h‐light/8‐h‐dark photoperiod at 21°C for Arabidopsis and for 7 days in incubators with 14‐h‐light/10‐h‐dark photoperiod at 28°C/24°C for rice. The seeds germinated properly in the plates from all the inhibitors, discarding any effect these compounds might have on seed germination. Plates were photographed, and the root length was measured with Fiji (Schindelin et al., 2012).

## CONFLICTS OF INTEREST

The authors report no conflicts of interest.

## AUTHOR CONTRIBUTIONS

**Maria Florencia Ercoli:** Root assays; data analysis. **Priscila Zonzini Ramos:** Cloning into expression vector; small‐scale test expression; data analysis. **Rashmi Jain:** Selection of kinases and Figure 1 (phylogenetic tree); data analysis. **Joseph Pilotte:** Protein production; DSF screening. Oliver Xiaoou Dong: Coordination of the de novo synthesis of the 129 rice kinase genes. **Ty Thompson:** Root assays. **Carrow I. Wells:** DSF screening; data analysis. **Jonathan M. Elkins:** Kinase domain analysis and expression construct design. **Aled M. Edwards:** Project conceptualization. **Rafael M. Couñago:** Manuscript writing; data analysis. **Pamela C. Ronald:** Project conceptualization; manuscript writing. **David H. Drewry:** Project conceptualization; manuscript writing; data analysis. All: Manuscript editing.

## Supporting information


**Figure S1.** Cloning and small‐scale test expression of LOC_Os06g16330 kinase domain (KD). (A) Agarose gel showing amplicons from positive clones amplified from bacterial colonies by PCR. Four different constructs were designed for this rice kinase. M: molecular weight marker (1 Kb Plus DNA Ladder, Invitrogen). (B) SDS‐PAGE analysis of eluted fractions obtained from small‐scale test expression in both BL21(DE3)‐R3‐pRARE2 (p) and BL21(DE3)‐R3‐lambda‐PPase (λ) strains. M: molecular weight marker (Precision Plus Protein Unstained Protein Standards, Bio‐Rad). (C) Liquid chromatography‐mass spectrometry (LC‐MS) analysis for LOC_Os06g16330 KD purified from small‐sale test expression (construct 3 – indicated in panel B). Deconvoluted mass/charge spectra are shown for proteins expressed in BL21(DE3)‐R3‐pRARE2 (top) and BL21(DE3)‐R3‐lambda‐PPase (bottom) strains. Expected and observed mass values are indicated. In addition to the correct mass, three phosphorylation states were noticed when the protein was not co‐expressed with Lambda Protein Phosphatase (top).
**Figure S2.** Test expression of all 129 rice protein kinases selected for this study. SDS‐PAGE analysis of (metal ion) affinity‐purified proteins obtained from small‐scale test expressions in both BL21(DE3)‐R3‐pRARE2 (p) and BL21(DE3)‐R3‐lambda‐PPase (λ) strains. Precision Plus Protein Unstained Protein Standards (Bio‐Rad) was used as a molecular weight marker. The presence of a band with the expected molecular weight indicates that the protein was successfully produced in a soluble manner. The protein expression level was estimated based on relative band intensity. Rice‐Plate‐1 / B08, B11 p and B11 λ illustrate respectively high, medium and low expression levels of soluble protein. The absence of a band with the expected molecular weight indicates that no soluble protein was detected (e.g., Rice‐Plate‐1 / A04 p and λ). Empty lanes represent failed test expressions and are identified in red (e.g., Rice‐Plate‐1 / B10 p and λ). Results for all test expressions are summarized in Supplemental Data Set 1. Red arrows indicate the 40 rice protein kinases that were further produced in large scale and screened using differential scanning fluorimetry (DSF) against 627 diverse kinase inhibitors.
**Figure S3.** Root growth phenotypes in Arabidopsis plants treated with human kinase inhibitors. (A) Root growth phenotype 6d after sowing of Col‐0 seedlings grown on 1xMS vertical plates with or without 1 μM of the selected kinase inhibitor showing a significant effect on primary root growth. (B) and (C) Primary root length (mm) 6d after sowing of Col‐0 seedlings grown on 1xMS vertical plates with different chemical treatments. In (B) plates were prepared with or without 1 μM of the selected kinase inhibitor. In (C) we used different combinations of two selected kinase inhibitors that are known to bind the rice orthologue of AtPSKR1 (#24 and #37, 1 μM) and PSK1 (100 nM). The data shown in (B) and (C) are a box and whisker plot combined with scatter plots, each dot indicates an individual measurement (n = 20–30).
**Figure S4.** Multiple sequence alignment showing sequence similarity between human and rice kinases hit by the same compounds. All 11 subdomains indicative of a protein kinase are highlighted with roman numerals above the alignment. BRAF Residues involved in binding compound RAF265 are marked with red stars above the alignment, excluding the invariant DFG motif and Lys‐Glu bridge which are involved in binding RAF265 but are highly conserved in all protein kinases, indicating that BRAF residues involved in binding RAF265 show a high degree of conservation across the 9 rice kinases that bind RAF265, suggesting that RAF265 is also an ATP‐competitive inhibitor of the rice kinases.

**Data S1.** Supporting Information

**Data S2.** Supporting Information

**Data Set S1.** Results from cloning and small‐cale test expression of the 129 rice protein kinases selected for this study. Detailed information of rice kinase constructs successfully cloned, such as target genes, construct IDs, construct regions (kinase domain boundaries), primer sequences, PCR fragment lengths and protein molecular weights are shown. Expression levels of protein observed from small‐scale test expression in both BL21(DE3)‐R3‐pRARE2 (pRARE2) and BL21(DE3)‐R3‐lambda‐PPase (λ‐PPase) strains are also indicated. Clones selected for scale‐up production were verified by DNA sequencing and the identity of the purified proteins was further confirmed by mass spectrometry (MS).

**Data Set S2.** DSF screening data for kinase inhibitors and rice kinases. Compounds are listed in column A, and kinases are listed in columns B to AD. Each row has melting temperature shift data for the compound in column A against each rice kinase screened

**Data Set S3.** Supporting Information

**Data Set S4.** Column A: List of kinase inhibits tested. Column B: #number of plant kinases stabilized by compound in DSF assay. Column C: Is the compound FDA approved?

## Data Availability

All data supporting the findings of this study are available within the paper and within the supporting information.
