# Supplementary material for: An open source plant kinase chemogenomics set
Source: Plant Direct. 2022 Nov 25;6(11):e460. doi: 10.1002/pld3.460 (PMC9694430; doi:10.1002/pld3.460)
Supplement: Supplementary file 1 — Figure S1. Cloning and small‐scale test expression of LOC_Os06g16330 kinase domain (KD). (A) Agarose gel showing amplicons from positive clones amplified from bacterial colonies by PCR. Four different constructs were designed for this rice kinase. M: molecular weight marker (1 Kb Plus DNA Ladder, Invitrogen). (B) SDS‐PAGE analysis of eluted fractions obtained from small‐scale test expression in both BL21(DE3)‐R3‐pRARE2 (p) and BL21(DE3)‐R3‐lambda‐PPase (λ) strains. M: molecular weight marker (Precision Plus Protein Unstained Protein Standards, Bio‐Rad). (C) Liquid chromatography‐mass spectrometry (LC‐MS) analysis for LOC_Os06g16330 KD purified from small‐sale test expression (construct 3 – indicated in panel B). Deconvoluted mass/charge spectra are shown for proteins expressed in BL21(DE3)‐R3‐pRARE2 (top) and BL21(DE3)‐R3‐lambda‐PPase (bottom) strains. Expected and observed mass values are indicated. In addition to the correct mass, three phosphorylation states were noticed when the protein was not co‐expressed with Lambda Protein Phosphatase (top). Figure S2. Test expression of all 129 rice protein kinases selected for this study. SDS‐PAGE analysis of (metal ion) affinity‐purified proteins obtained from small‐scale test expressions in both BL21(DE3)‐R3‐pRARE2 (p) and BL21(DE3)‐R3‐lambda‐PPase (λ) strains. Precision Plus Protein Unstained Protein Standards (Bio‐Rad) was used as a molecular weight marker. The presence of a band with the expected molecular weight indicates that the protein was successfully produced in a soluble manner. The protein expression level was estimated based on relative band intensity. Rice‐Plate‐1 / B08, B11 p and B11 λ illustrate respectively high, medium and low expression levels of soluble protein. The absence of a band with the expected molecular weight indicates that no soluble protein was detected (e.g., Rice‐Plate‐1 / A04 p and λ). Empty lanes represent failed test expressions and are identified in red (e.g., Rice‐Plate‐1 / B10 p and [file PLD3-6-e460-s004.docx]

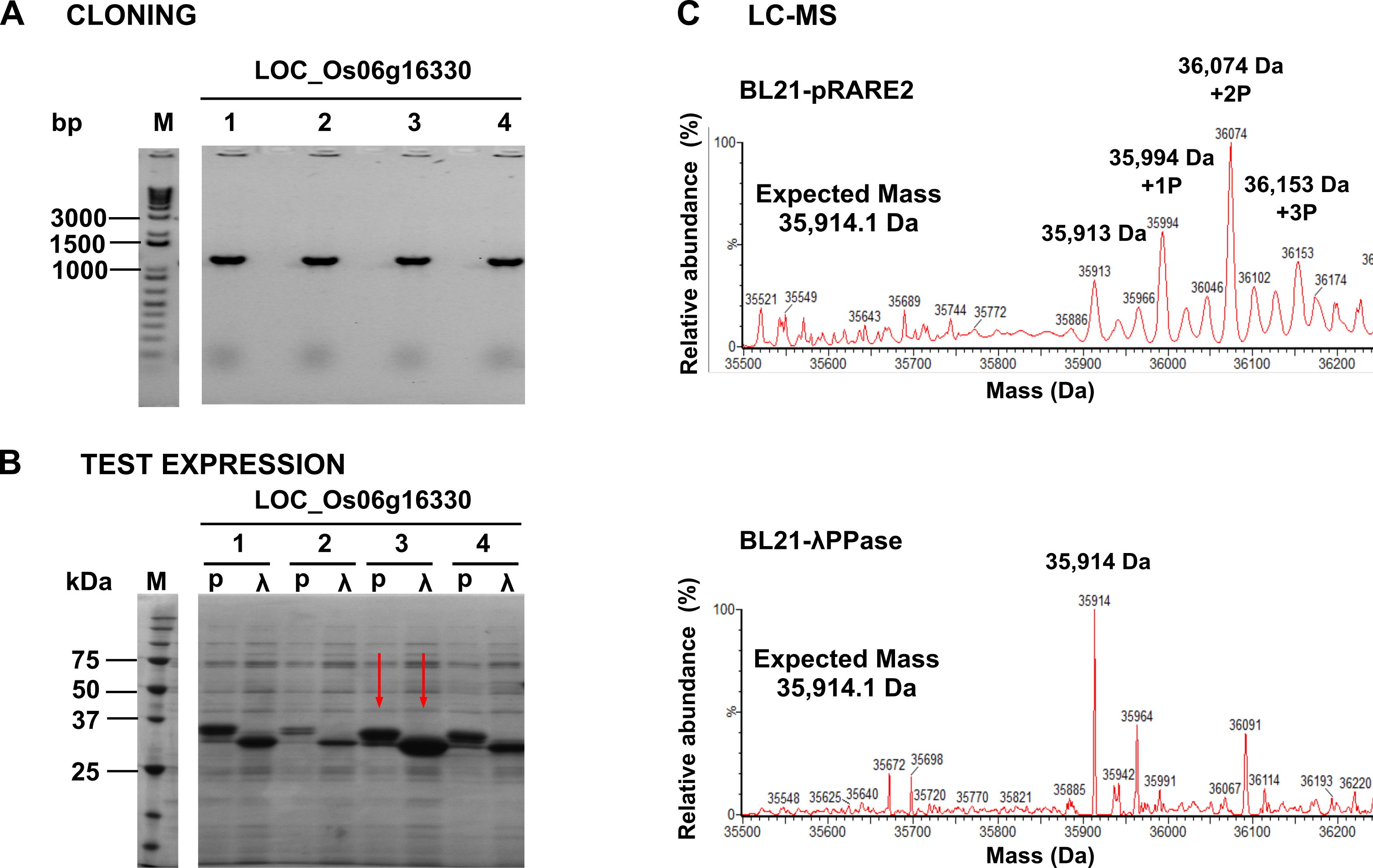


**Supplemental Figure S1.** **Cloning and small-scale test expression of LOC_Os06g16330 kinase domain (KD).**

(**A**) Agarose gel showing amplicons from positive clones amplified from bacterial colonies by PCR. Four different constructs were designed for this rice kinase. M: molecular weight marker (1 Kb Plus DNA Ladder, Invitrogen). (**B)** SDS-PAGE analysis of eluted fractions obtained from small-scale test expression in both BL21(DE3)-R3-pRARE2 (p) and BL21(DE3)-R3-lambda-PPase (λ) strains. M: molecular weight marker (Precision Plus Protein Unstained Protein Standards, Bio-Rad). (**C)** Liquid chromatography-mass spectrometry (LC-MS) analysis for LOC_Os06g16330 KD purified from small-sale test expression (construct 3 – indicated in panel B). Deconvoluted mass/charge spectra are shown for proteins expressed in BL21(DE3)-R3-pRARE2 (top) and BL21(DE3)-R3-lambda-PPase (bottom) strains. Expected and observed mass values are indicated. In addition to the correct mass, three phosphorylation states were noticed when the protein was not co-expressed with Lambda Protein Phosphatase (top).


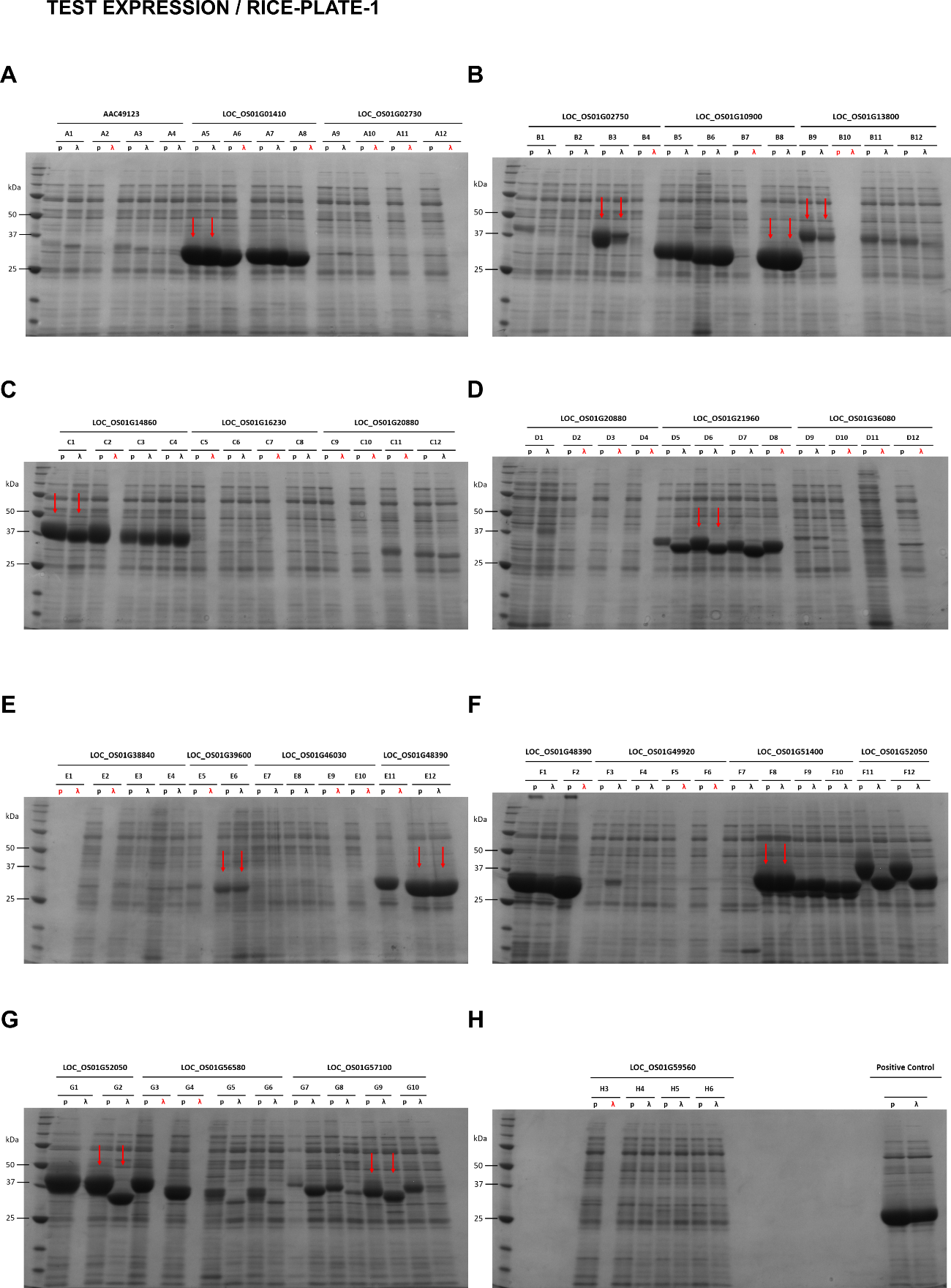


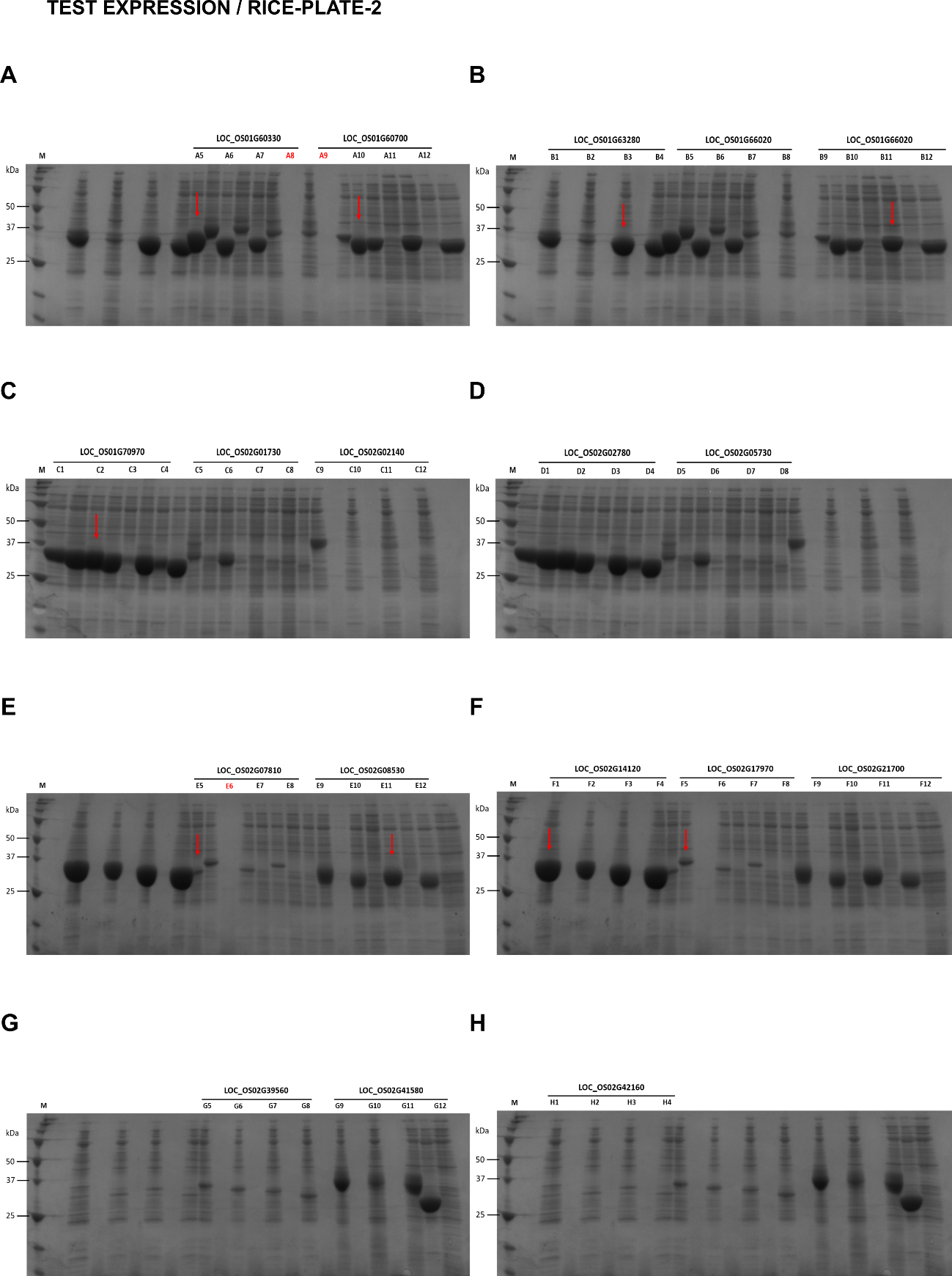


*: Rice-Plate-2 test expression results are available only for BL21(DE3)-R3-pRARE2 strain.


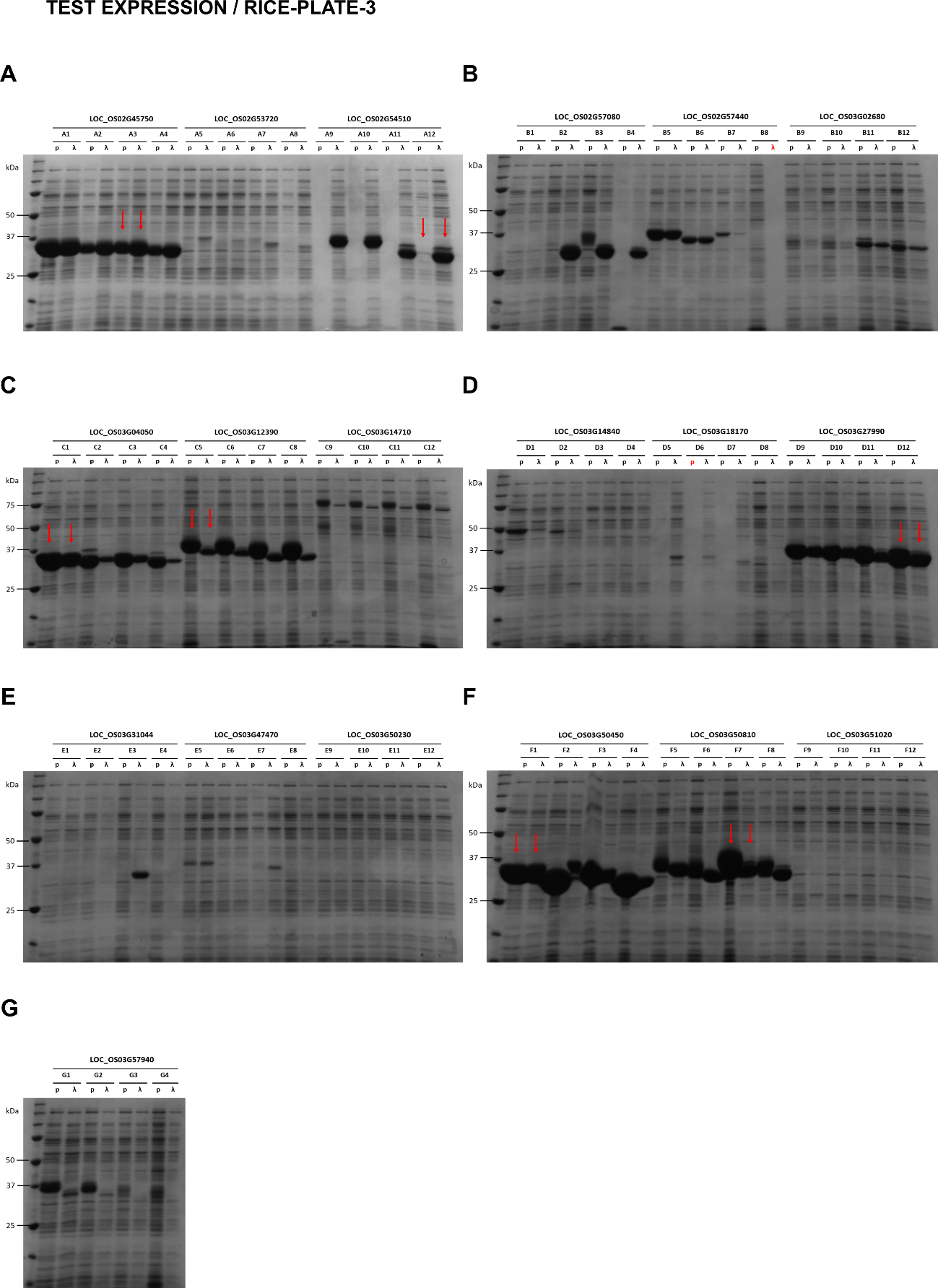


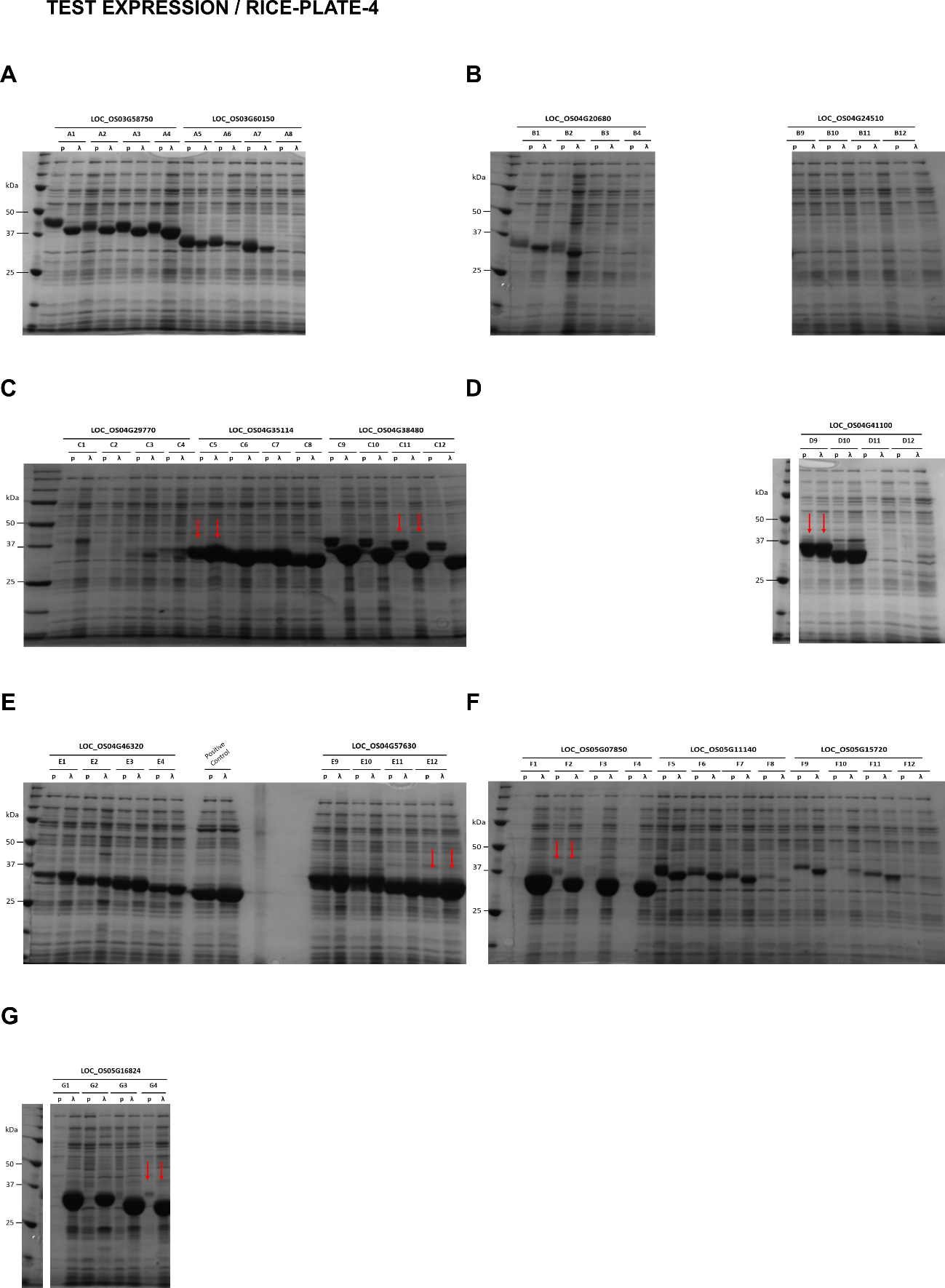


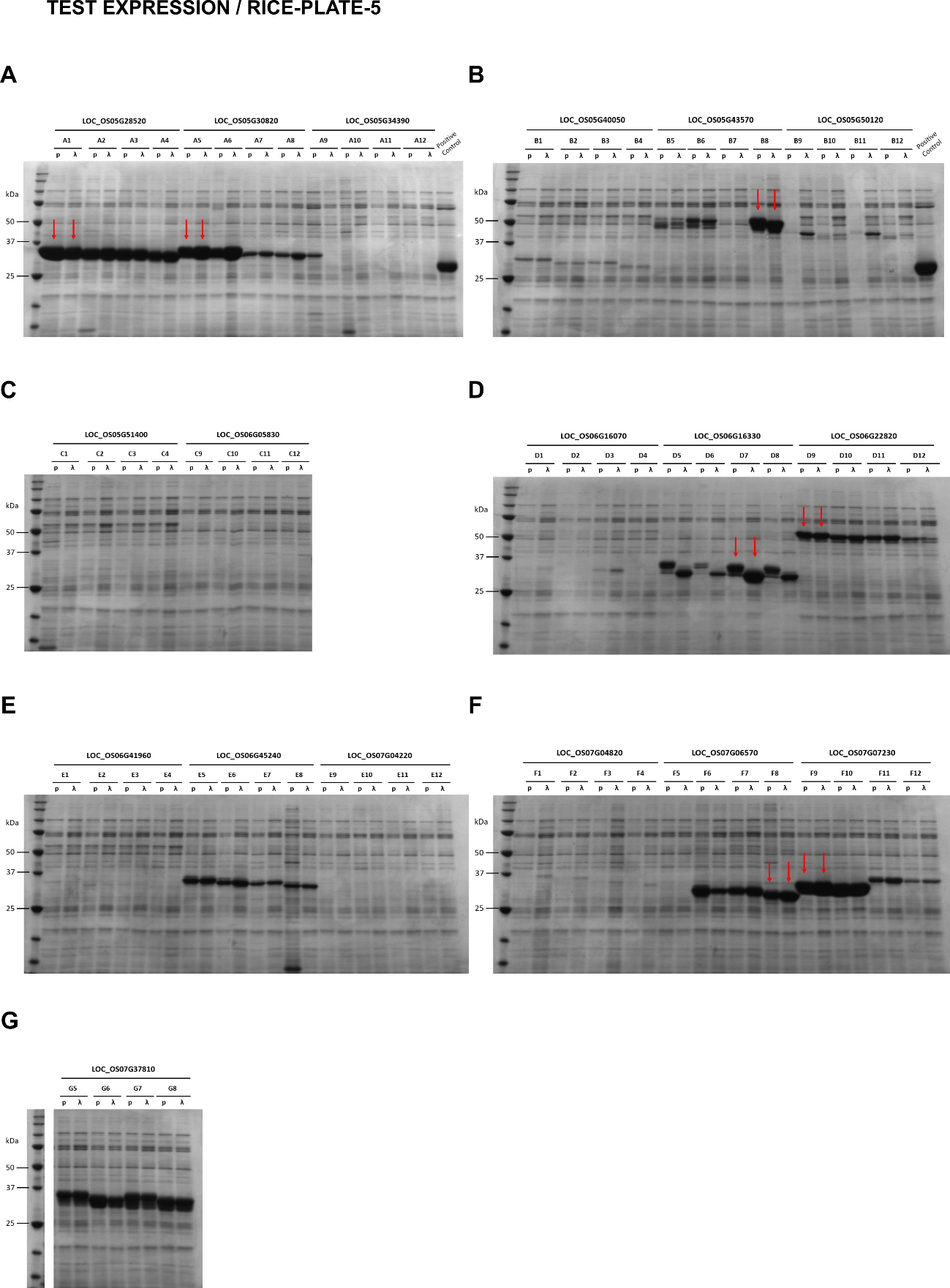


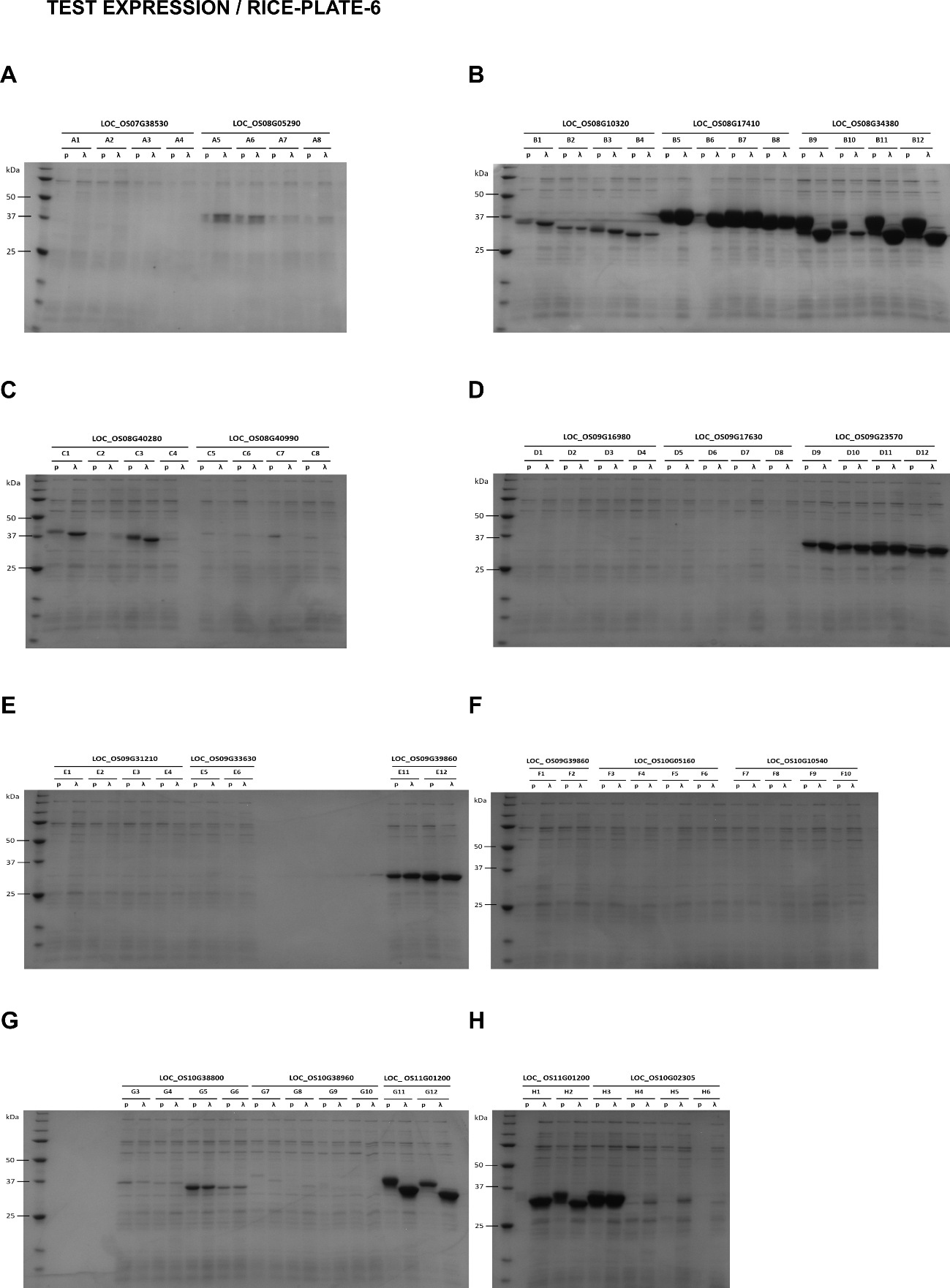


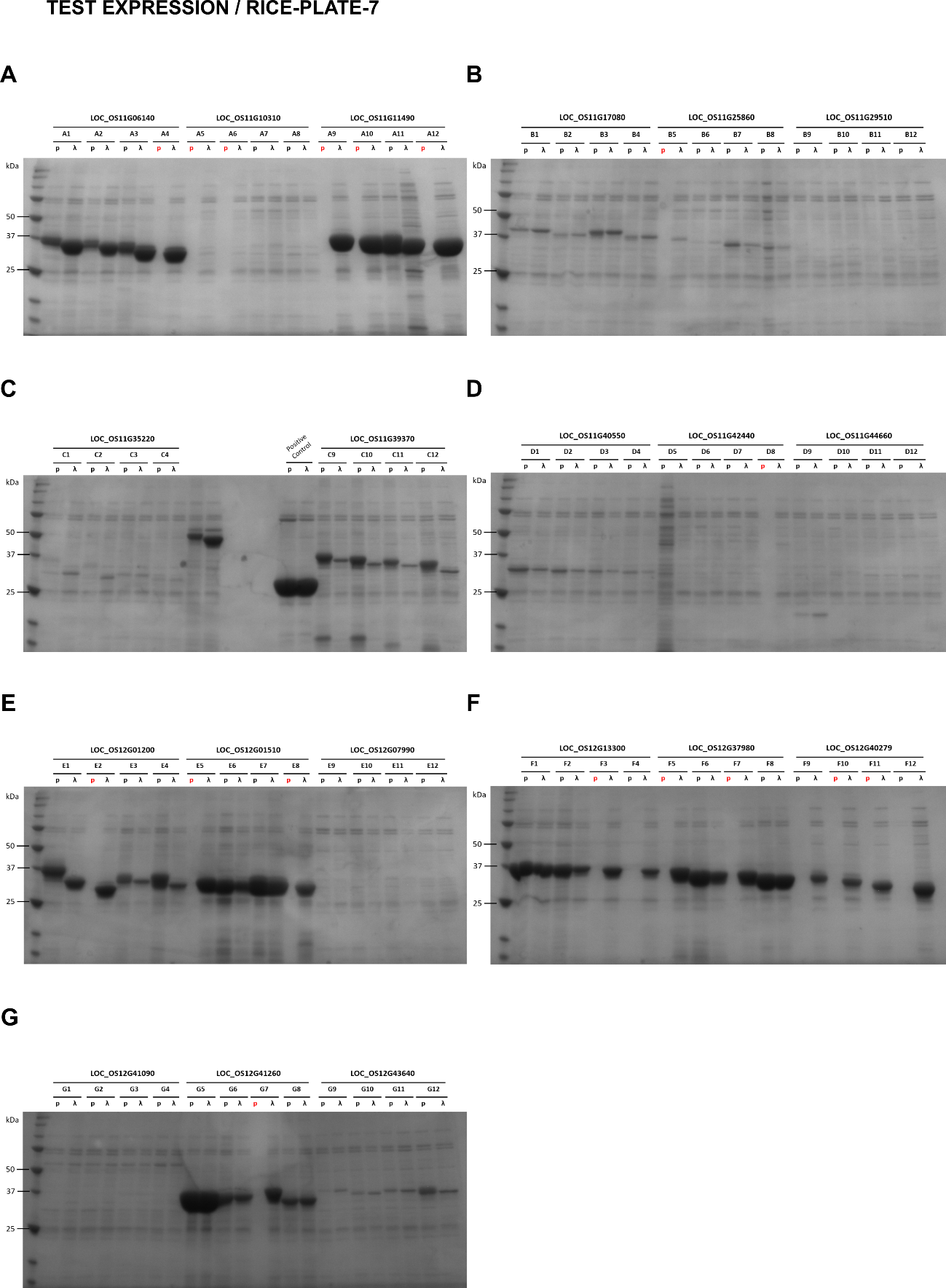


**Supplemental Figure S2. Test expression of all 129 rice protein kinases selected for this study.**

SDS-PAGE analysis of (metal ion) affinity-purified proteins obtained from small-scale test expressions in both BL21(DE3)-R3-pRARE2 (p) and BL21(DE3)-R3-lambda-PPase (λ) strains. Precision Plus Protein Unstained Protein Standards (Bio-Rad) was used as a molecular weight marker. The presence of a band with the expected molecular weight indicates that the protein was successfully produced in a soluble manner. The protein expression level was estimated based on relative band intensity. Rice-Plate-1 / B08, B11 p and B11 λ illustrate respectively high, medium and low expression levels of soluble protein. The absence of a band with the expected molecular weight indicates that no soluble protein was detected (e.g., Rice-Plate-1 / A04 p and λ). Empty lanes represent failed test expressions and are identified in red (e.g., Rice-Plate-1 / B10 p and λ). Results for all test expressions are summarized in Supplemental Data Set 1. Red arrows indicate the 40 rice protein kinases that were further produced in large scale and screened using differential scanning fluorimetry (DSF) against 627 diverse kinase inhibitors.


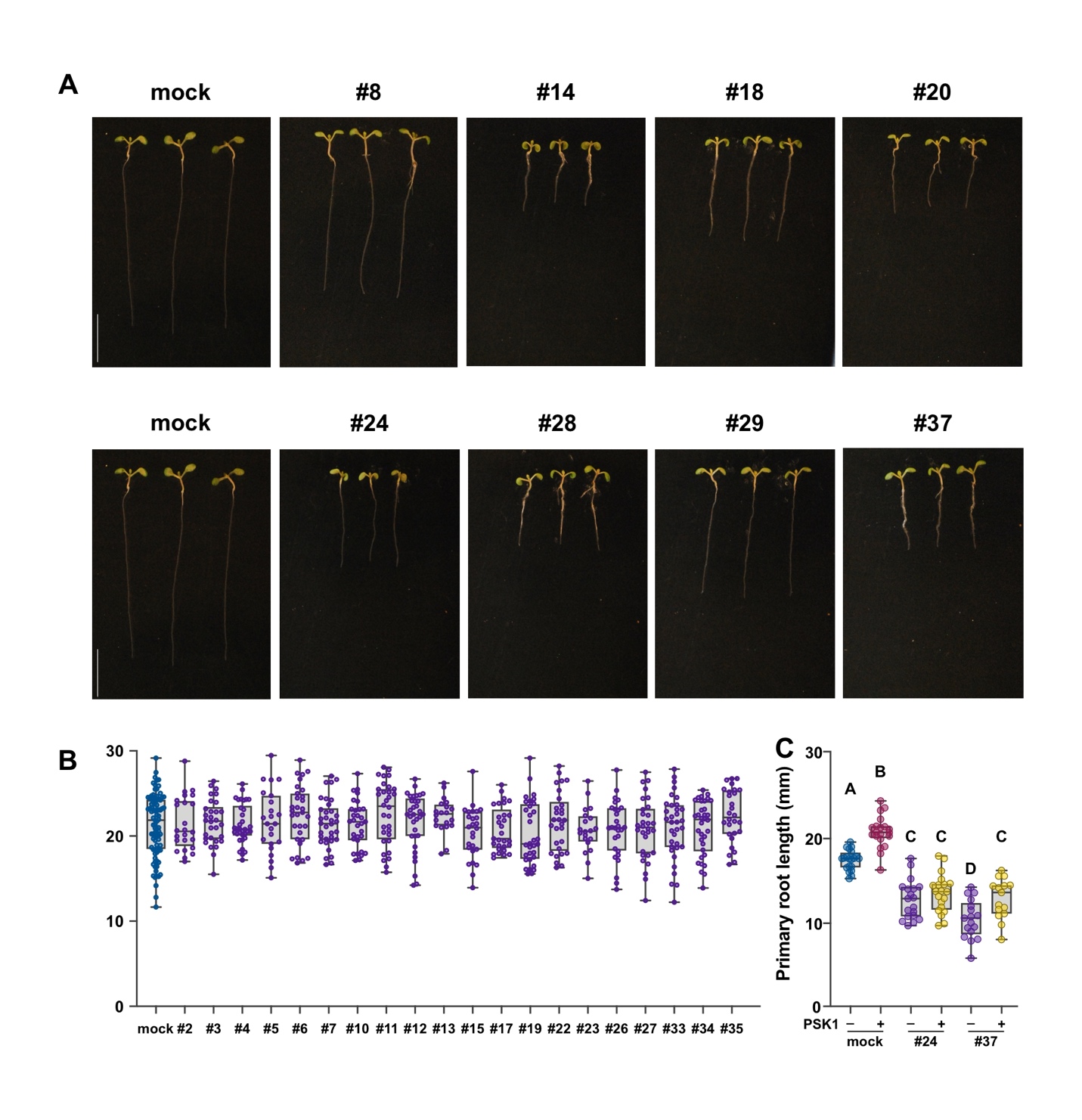


**Supplementary Figure S3. Root growth phenotypes in Arabidopsis plants treated with human kinase inhibitors.**

**(A)** Root growth phenotype 6d after sowing of Col-0 seedlings grown on 1xMS vertical plates with or without 1 µM of the selected kinase inhibitor showing a significant effect on primary root growth. **(B)** and **(C)** Primary root length (mm) 6d after sowing of Col-0 seedlings grown on 1xMS vertical plates with different chemical treatments. In **(B)** plates were prepared with or without 1 µM of the selected kinase inhibitor. In **(C)** we used different combinations of two selected kinase inhibitors that are known to bind the rice orthologue of AtPSKR1 (#24 and #37, 1 µM) and PSK1 (100 nM). The data shown in **(B)** and **(C)** are a box and whisker plot combined with scatter plots, each dot indicates an individual measurement (n=20-30).

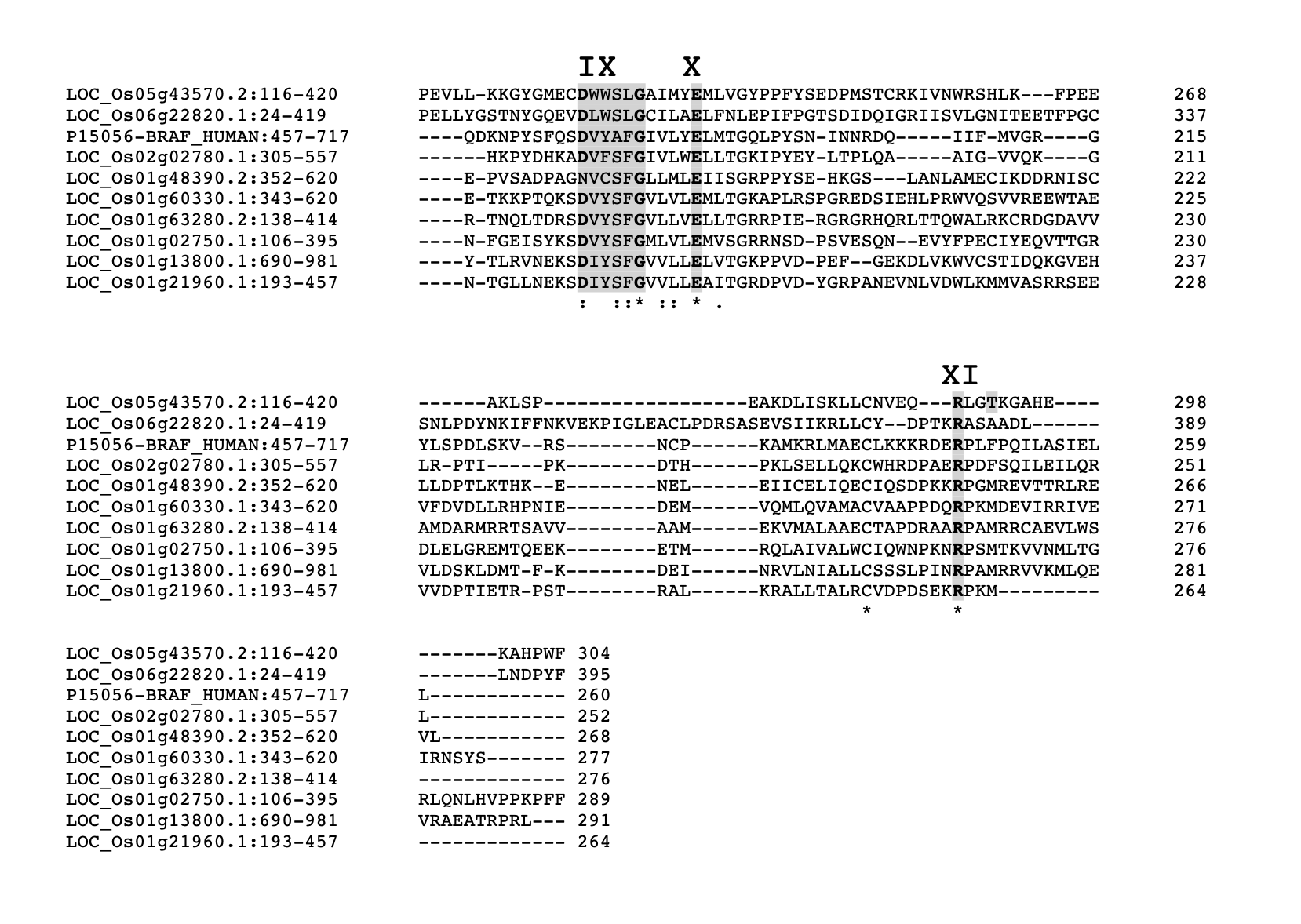


**Supplementary Figure 4.  Multiple sequence alignment showing sequence similarity between human and rice kinases hit by the same compounds.**All 11 subdomains indicative of a protein kinase are highlighted with roman numerals above the alignment. BRAF Residues involved in binding compound RAF265 are marked with red stars above the alignment, excluding the invariant DFG motif and Lys-Glu bridge which are involved in binding RAF265 but are highly conserved in all protein kinases, indicating that BRAF residues involved in binding RAF265 show a high degree of conservation across the 9 rice kinases that bind RAF265, suggesting that RAF265 is also an ATP-competitive inhibitor of the rice kinases.
