# Supplementary material for: An open source plant kinase chemogenomics set
Source: Plant Direct. 2022 Nov 25;6(11):e460. doi: 10.1002/pld3.460 (PMC9694430; doi:10.1002/pld3.460)
Supplement: Supplementary file 2 — Data S1. Supporting Information [file PLD3-6-e460-s006.docx]

Dear Rubén Rellán-Álvarez and Hsou-min Li,

Thank you for giving us the opportunity to submit a revised draft of our manuscript titled “An open source plant kinase chemogenomics set” to *Plant Direct*. We appreciate the time and effort that you and the reviewers have dedicated to reviewing this MS. We have addressed the recommended changes and have highlighted the changes within the manuscript.

Here is a point-by-point response to the reviewers’ comments and concerns.

----------------------------------------------------------------------------: 


Both reviewers have raised issues about the validation of kinase activity and have suggested further validation using mutants or in-vitro assays. These are similar comments raised by the reviewers in Plant Cell before transferring to Plant Direct. I don't think experimental validation is necessary for publication in Plant Direct. Your work is a phenomenal resource for the plant community to winnow down candidate inhibitors. But please, clarify that further experimentation is necessary to prove inhibition by candidate inhibitors on a one-by-one basis.

*We have clarified that point in the revised discussion.*

---------------------------------------------------------------------------- Reviewer comments: 
Reviewer #1: 

The manuscript by Ercoli et al describes a screening of a large set of rice kinases and the interaction with chemical kinase inhibitors based on human kinases information. This kind of large screenings are of key importance finding novel kinase inhibitors which may be useful for future functional analysis. The manuscript is presented in an amenable form to be understandable, however it lacks of information which is useful for readers in this field. I have some suggestions to better the manuscript. 

1. In the recombinant production of selected rice protein kinases, I wonder if authors confirmed the identity of the interest proteins by Western blot using an Anti-HIS antibody at least for a representative number of them.

*In this MS we chose to confirm the identity of the IMAC-purified proteins using mass spectrometry (LC-MS). In the context of this work, confirming the identity of the purified proteins via LC-MS is advantageous compared to the use of Western blots and anti-His antibodies. First, LC-MS provides the intact mass of the purified protein with great precision, and this value can easily be confirmed by comparing with the predicted mass encoded by the DNA sequence construct. Secondly, LC-MS provides information on the phosphorylation status of the sample – which is of particular importance for protein kinases. Please note that all DNA constructs were confirmed by sequencing.*

2. A SDS-PAGE figure with all the 40 purified proteins would be useful to have an estimate of the efficiency of purification of plant protein kinases in E. coli.

*. Following the referee’s suggestion, we prepared a Supplemental Figure (S2) showing the results (SDS-PAGE) of small-scale expression/purification of all 129 rice protein kinases reported in this work. In this same figure, we indicate the 40 rice protein kinases selected for further characterization (large scale production and ligand screen by DSF).*

3. In the ligand identification section, information in the Supp. Material 1 about the DSF screening data for kinase inhibitors and rice kinases is key for understanding the manuscript. I recommend to present this information in a graphic way showing the inhibitors with significant melting temperature shift for corresponding protein kinases. As well as for data presented in Supp. Material 2 with rice kinase inhibitors tested.

*We agree that understanding these data are important for understanding the manuscript as a whole. In figure 2, we have shared 3 ways one can look at the data, which will help the reader understand the data. In the supplemental material, we have provided all of the thermal shift screening data. In the excel spreadsheet that provides a complete data matrix (compounds vs kinases), we have highlighted values that represent a significant temperature shift in yellow to quickly highlight to the reader compounds of interest. Based on the reviewer’s suggestion, we have added another excel file to the supplemental information package (Supplemental Data Set 3). Here we have sorted the thermal shift data for each kinase separately, allowing the significant hit compounds to rise to the top for each kinase. Again, these are highlighted in yellow to draw attention to them, but now one does not have to sift through the large spreadsheet to identify the names of the compound hits. There is no simple way to visualize this very large data matrix in a clear intuitive way for all readers, so we believe the combination of these two spreadsheets will provide the reader with what you are looking for.*

4. Thermal-stability assay by Differential Scanning Fluorimetry (DSF) assay is a good approach to identify ligands, however, this study would be more robust if authors could test experimentally affected kinase activity of some representative rice kinases by the chemical inhibitors identified. They could make in vitro kinase assays with and without inhibitors, ideally with kinases shown to have an effect in root development.

*We agree that for kinases, the DSF approach has proven to be a robust technique utilized in many labs as a way to identify ligands for kinases, including understudied kinases for which very little is known. We have not followed up on the individual hits and agree that further experimentation is necessary to prove the inhibition of the candidate enzymes for these plant kinases. We have highlighted this point in the discussion of our revised MS. Our focus was the strategy and the winnowing of compounds to narrow the field of view for further work. Follow-up work in our labs or any lab pursuing this approach should include an investment in enzymology as a next step to add further confirmation linking phenotype to target(s).*

5. In the last experiment about the effect of inhibitors on the root phenotype in Arabidopsis and in rice, conclusions are quite speculative, since the authors are not using mutants effected in the function of the specific kinases they are testing. The effect of inhibitors may be on many active kinases in the developmental stage tested. They should evaluate the effect of the inhibitors in at least one mutant with no function of the kinase in study. 
*We agree with this comment. We have now tested the effect of two inhibitory compounds that bind the AtPSKR1 orthologue in a mutant line that has two paralogous receptor kinases involved in peptide growth-promoting signaling (Atpskr1, Atpskr2) knocked out. The double mutant seedling produced shorter primary roots than the control, a phenotype that cannot be complemented by exogenous PSK treatment because this mutant is PSK insensitive. When this mutant was treated with compound #24, we saw no further reduction in primary root length (Figure 3C). This result supports the previous observation that PSK exogenous treatment when combined with inhibitory compound #24, does not induce primary root elongation in control plants (Supplemental Figure S3C). Compound #37, on the other hand, produces a slight inhibition of triple mutant root growth, though this inhibition is not as strong as that observed when treating control plants.*

Reviewer #2: 

Thank-you for asking my opinion on this nice study, which I enjoyed reading very much. I think this will be a valuable toolkit for plant kinase research and it sets a great framework for future study. I think the experiments are carefully conducted, well controlled, and adequately analyzed. The paper is well written and data well presented. 

Conclusions are mostly in line with the results, however I was surprised that there was no experimental evidence provided to indicate kinase inhibition in vitro. I think it should be relatively straightforward to choose a few representative kinases that have autophosphorylation activity and assess whether a subset of inhibitors (perhaps 1-2 of the compounds used in Fig 3) can decrease activity in vitro. That said, the authors do not claim that these compounds necessarily inhibit plant kinases - rather carefully phrasing to say that they bind kinases and therefore deserve further study. I thus leave it to the editor and authors to decide if in vitro kinase assays are needed. 

Perhaps this is a formatting issue, but I would have liked to read a Discussion section that puts this toolkit into a broader perspective, perhaps with some figures to show expected binding pockets. Although this is well described in the text, it would be useful for the community. 

Overall, well done and thank-you for this significant body of work.

*We agree with this and have incorporated a Discussion section into the manuscript.*
